# Comparing pentafecta outcomes between nerve sparing and non nerve sparing robot-assisted radical prostatectomy in a propensity score-matched study

**DOI:** 10.1038/s41598-023-43092-z

**Published:** 2023-09-22

**Authors:** Tanan Bejrananda, Kiyoshi Takahara, Dutsadee Sowanthip, Tomonari Motonaga, Kota Yagi, Wataru Nakamura, Masanobu Saruta, Takuhisa Nukaya, Masashi Takenaka, Kenji Zennami, Manabu Ichino, Hitomi Sasaki, Makoto Sumitomo, Ryoichi Shiroki

**Affiliations:** 1 Division of Urology, Department of Surgery, Faculty of Medicine, Prince of Songkla University, Hat Yai, 90110 Songkhla Thailand; 2 Department of Urology, Fujita Health University School of Medicine, 1-98 Dengakugakubo, Kutsukakecho, Toyoake, Aichi 470-1192 Japan; 3 Division of Urology, Department of Surgery, Faculty of Medicine, Chulalongkorn University, King Chulalongkorn Memorial Hospital, The Thai Red Cross Society, Bangkok, Thailand

**Keywords:** Oncology, Urology

## Abstract

Pentafecta (continence, potency, cancer control, free surgical margins, and no complications) is an important outcome of prostatectomy. Our objective was to assess the pentafecta achievement between nerve-spring and non-nerve-sparing robot-assisted radical prostatectomy (RARP) in a large single-center cohort. The study included 1674 patients treated with RARP between August 2009 and November 2022 to assess the clinical outcomes. Cox regression analyses were performed to evaluate the prognostic significance of RARP for pentafecta achievement, and 1:1 propensity score matching (PSM) was performed between the nerve-sparing and non-nerve-sparing to test the validity of the results. Pentafecta definition included continence, which was defined as the use of zero pads; potency, which was defined as the ability to achieve and maintain satisfactory erections or ones firm enough for sexual activity and sexual intercourse. The biochemical recurrence rate was defined as two consecutive PSA levels > 0.2 ng/mL after RARP; 90-day Clavien–Dindo complications ≤ 3a; and a negative surgical pathologic margin. The median follow-up period was 61.3 months (IQR 6–159 months). A multivariate Cox regression analysis demonstrated that pentafecta achievement was significantly associated with nerve-sparing (NS) approach (1188 patients) (OR 4.16; 95% CI 2.51–6.9), *p* < 0.001), unilateral nerve preservation (983 patients) (OR 3.83; 95% CI 2.31–6.37, *p* < 0.001) and bilateral nerve preservation (205 patients) (OR 7.43; 95% CI 4.14–13.36, *p* < 0.001). After propensity matching, pentafecta achievement rates in the NS (476 patients) and non-NS (476 patients) groups were 72 (15.1%) and 19 (4%), respectively. (*p* < 0.001). NS in RARP offers a superior advantage in pentafecta achievement compared with non-NS RARP. This validation study provides the pentafecta outcome after RARP associated with nerve-sparing in clinical practice.

## Introduction

Prostate cancer (PCa) is the second most common cancer in men^1^. Radical prostatectomy (RP) is the mainstay of treatment for localized PCa. RP aims to accomplish the "pentafecta" by simultaneously ensuring the preservation of sexual potency and urinary continence, achieving negative surgical margins, avoiding surgical complications, and effectively controlling cancer after the procedure^2^. Nerve sparing (NS) is a key factor in improving continence and sexual outcomes. To maintain sexual potency and urinary continence, avoiding resection of the neurovascular bundle during NS RP^3–5^. However, NS may increase the rate of positive surgical margins during RP, which in turn may increase biochemical recurrence (BCR)^6^. Therefore, a delicate balance exists between conserving the neurovascular bundle through tissue preservation and the potential risk of encountering positive surgical margins (PSM).

PSM indicates incomplete tumor removal and is believed to be associated with a poor prognosis. Nonetheless, the significance of PSM remains contentious, with 27–44% of patients experiencing BCR, 6.8–24% of patients exhibiting systemic progression, and 0.8–3.7% of patients encountering PCa-related mortality over a follow-up period of 7–13 years^7,8^. Currently, RP alone is not enough to induce biochemical remission. Therefore, multiparametric evaluation methods such as trifecta are being developed, which assesses biochemical remission, continence, and erectile function. An improvement over the trifecta is the pentafecta, which additionally evaluates postoperative complications and surgical margin infiltration^9^.

The most favorable combined outcomes after RARP may confer a stable or even improved quality of life; however, up to one-third of patients may experience deterioration. This warrants further investigation on how to capture the underlying cause and address and potentially solve these perceived negative effects despite successful RARP^10^. Therefore, to address this unmet need, we aimed to analyze the pentafecta achievement rate according to the NS procedure in RARP and its impact on patient outcomes. We performed a propensity-matched analysis to limit the impact of selection bias on survival outcomes.

## Methods

### Study design

We included patients who underwent RARP as the primary treatment for localized and locally advanced prostate cancer between August 2009 and November 2022 at the Fujita Health University Hospital. Clinical variables were evaluated, and all cases were defined according to the D'Amico risk stratification system^11^. Clinical staging was performed using the unified TNM classification^12^. The criteria for nerve sparing were as follows-complete: non palpable disease with < 3 cores involvement on prostate biopsy; partial: non palpable disease with < 4 cores involvement on prostate biopsy; none: clinically palpable disease with ≥ 4 cores involvement on prostate biopsy and intraoperative visual cues of locally advanced disease (loss of dissection planes, focal bulge of prostatic capsule). The number of pads used daily at three and six months after RARP was checked to assess urinary continence recovery.

This study was approved by the Ethics Committee of the Fujita Health University Hospital (Approval No. HM19-265), and was performed in accordance with the ethical standards laid down in the most recent version of the Declaration of Helsinki.

### Pentafecta definition

Continence was defined as the use of “zero pads”. The number of pads used daily at three and six months after RARP was checked to assess urinary continence recovery. Potency is defined as the ability to achieve and maintain satisfactory erections or a firmness that is sufficient for sexual activity or sexual intercourse. The schedule after RARP consisted of a PSA assay every 3 months for the first 2 years, every 6 months for the following 3 years, and annually thereafter. The biochemical recurrence (BCR) rate was defined as two consecutive PSA levels > 0.2 ng/mL after RARP^2^. Absence of major perioperative complications (only presence of 90-day Clavien–Dindo complications (CDC) ≤ 3a)^13^, negative surgical margins. Only patients who successfully met all criteria were considered to have reached pentafecta.

### Statistical analyses

Statistical methods for clinical variables and definition of outcomes. A review of retrospectively obtained clinical data from electronic medical records was conducted. Patient information was anonymized and de-identified prior to the analysis. For each group, descriptive statistics were used to summarize the clinical presentation (age at diagnosis, biopsy GS grade, PSA level at diagnosis (PSA), and clinical T stage by MRI). Continuous variables are presented as medians (ranges) and categorical variables as numbers (percentages). The Mann–Whitney U-test was performed to determine the statistical significance of continuous variables between the NS RARP and non-NS RARP groups, while the chi-square test or Fisher’s exact test was used for categorical variables^14^.

**Figure 1:**
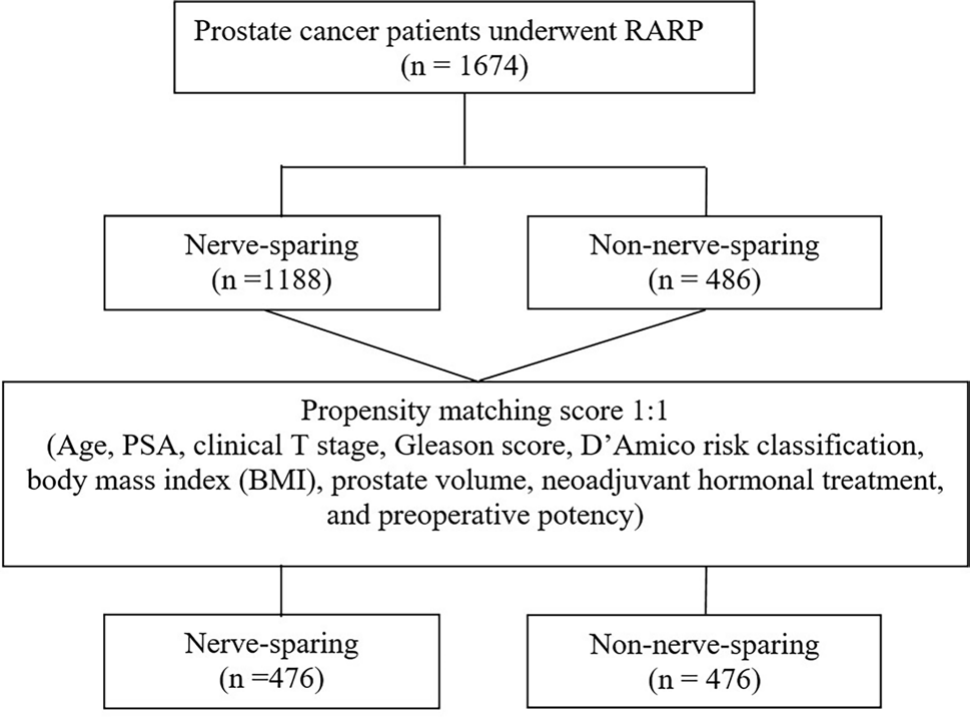
A flowchart with summary of patient enrollment and propensity score matching.

Propensity score (PS) matching analysis was performed to reduce selection bias in this retrospective study, achieving a greater comparison between the two groups. PS was calculated using a logistic regression model, and the covariates entered into the PS matching model were as follows: age at diagnosis, PSA level, clinical T stage, Gleason score, D’Amico classification, body mass index (BMI), prostate volume, neoadjuvant hormonal treatment, and preoperative potency. PS matching was performed using the 1:1 matching method. (Fig. 1) Univariate logistic regression was used to identify factors associated with pentafecta achievement. Statistically significant variables were included in the multivariate analysis of pentafecta achievement. Statistical significance was set at *p* < 0.05 was considered. All statistical analyses were performed using R version 4.3.0.

### Ethics approval and consent to participate

This study was approved by the Ethics Committee of the Fujita Health University Hospital (approval no. HM19-265). All procedures were performed in accordance with the ethical standards of the institutional and national research committees and the 1964 Declaration of Helsinki and its later amendments or comparable ethical standards. Informed consent was obtained from all patients according to the local ethics policy for retrospective analysis of our own anonymised clinical data.

## Results

### Demographic characteristics (entire cohort)

We enrolled 1674 consecutive patients with clinically localized PCa who underwent RARP performed by expert surgeons at Fujita Health University Hospital between August 2009 and November 2022. The median follow-up period was 61.3 months (interquartile range (IQR): 6–159 months). In all cohorts, pentafecta achievement was 14.9%. The baseline characteristics of the cohort and important operative outcomes are summarized in Table 1.

**Table 1:** Clinicopathologic characteristics of 1674 patients with PCa treated with RARP according to nerve approach and clinical outcomes.

Variables	Full cohort (n = 1674, 100%)
Age, years, median, (IQR)	67 (63,71)
Age, number	
< 65 years	557 (33.3)
> = 65 years	1115 (66.6)
Unknown	2 (0.1)
PSA (ng/mL), median, (IQR)	7.5 (5.5,10.7)
PSA (ng/mL)	
< 10	1176 (70.3)
10–19.99	385 (23.0)
> = 20	110 (6.4)
Unknown	3 (0.3)
Clinical T stage	
1-2b	706 (42.1)
2c	584 (34.9)
3–4	380 (22.7)
Unknown	4 (0.3)
Gleason score	
6	388 (23.2)
7	853 (49.9)
8–10	429 (25.6)
Unknown	4 (0.3)
D’Amico risk classification	
High	688 (41.1)
Intermediate	756 (45.2)
Low	226 (13.4)
Unknown	4 (0.3)
BMI (kg/m^2^), median, (IQR)	23.5 (21.8,25.5)
BMI group (kg/m^2^)	
< 18.5	38 (2.3)
18.5–22.9	637 (38.1)
23–24.9	472 (28.2)
25–29.9	484 (28.9)
> = 30	33 (2)
Unknown	10 (0.6)
Prostate volume preoperative group (cm^3^)	
< 35	864 (51.6)
> = 35	710 (42.4)
Unknown	100 (6.0)
Neoadjuvant treatment	
None	1067 (63.7)
ADT	166 (9.9)
Anti-A	177 (10.6)
NACHT	244 (14.6)
Unknown	20 (1.2)
Preoperative potency	
No erection	669 (40)
Erection	528 (31.5)
Penetration	447 (26.7)
Unknown	30 (1.8)
Operative time, min, (IQR)	171 (145,204)
Console time, min, (IQR)	119 (99,147)
Blood loss, mil, (IQR)	205.5 (125,350)
Nerve-sparing	
Yes	1188 (71)
No	484 (29)
Pathologic T stage	
1-2b	388 (23.2)
2c	1010 (60.3)
3–4	276 (16.5)
Surgical margin	
Negative	1333 (79.6)
Positive	341 (20.4)
Biochemical recurrence	
Absent	1500 (89.6)
Present	161 (9.6)
Unknown	13 (0.8)
Postoperative potency	
No erection	1140 (68.1)
Erection	354 (21.2)
Penetrate	111 (6.6)
Unknown	69 (4.1)
90 days complications according to the Clavien–Dindo classifcation	
< = 3a	1610 (96.2)
> = 3b	57 (3.4)
Unknown	7 (0.4)
Pentafecta achievement	
Yes	249 (14.9)
No	1353 (80.8)
Unknown	72 (4.3)

*PSA* Prostatic specific antigen; *IQR* Interquartile range; *BMI* Body mass index; *ADT* Androgen deprivation therapy; *Anti-A* Antiandrogen; *NACHT* Neoadjuvant combined hormonal therapy; *CDC* Clavien–Dindo-complications.

### Univariate and multivariate analysis of pentafecta achievement

Cox proportional hazards models with 95% confidence intervals were used to analyze the relationship between clinical variables and pentafecta achievement in RARP. We also performed multivariate Cox regression analysis for clinical variables with *p* < 0.05. In the univariate analysis, nerve-sparing (OR 5.9; 95% CI 3.65–9.54); *p* < 0.001), nerve-sparing technique include unilateral (OR 4.89; 95% CI 3.7–7.96); *p* < 0.001) and bilateral approach (OR 11.93; 95% CI 6.93–20.55; *p* < 0.001), age lower than 65 years (OR 2.22; 95% CI 1.69–2.92; *p* < 0.001), PSA < 10 ng/mL (OR 3.0; 95% CI 0.84–6.56); *p* < 0.001), Gleason score 6 (OR 2.91; 95% CI 1.81–4.69); *p* < 0.001), lower clinical T stage (1-2b) (OR 4.43; 95% CI 1.79–10.99); *p* < 0.001), lower pathological T stage (1-2b) (OR 4.48; 95% CI 2.77–8.45); *p* < 0.001), lower D’Amico risk classification (low risk) (OR 2.36; 95% CI 1.6–3.48); *p* < 0.001), were associated with pentafecta achievement. When multivariate analysis was performed, nerve-sparing (OR 4.16; 95% CI 2.51–6.9; *p* < 0.001), unilateral nerve-sparing (OR 3.83; 95% CI 2.31–6.37); *p* < 0.001), bilateral nerve-sparing (OR 7.43; 95% CI 4.14–13.36; *p* < 0.001) and Gleason score 6 (OR 1.86; 95% CI 1.08–3.22; *p* = 0.026), were the independent factors for pentafecta achievement. (Table 2).

**Table 2:** Univariate and multivariate Cox’s proportional hazard regression models to predict pentafecta achievement.

Variable	Univariate		Multivariate	
	OR (95%CI)	*p*-value	OR (95%CI)	*p*-value
Nerve-sparing				
No	Ref		Ref	
Yes	5.9 (3.65,9.54)	< 0.001	4.16 (2.51,6.9)	< 0.001
Nerve-sparing technique				
None	Ref	< 0.001	Ref	< 0.001
Unilateral	4.89 (3.7,7.96)	< 0.001	3.83 (2.31,6.37)	< 0.001
Bilateral	11.93 (6.93,20.55)	< 0.001	7.43 (4.14,13.36)	< 0.001
Age group				
> = 65	Ref			
< 65	2.22 (1.69,2.92)	< 0.001	1.25 (0.91,1.71)	0.166
PSA at diagnosis				
> = 20	Ref		Ref	
10–19.99	1.91 (0.84,4.37)	0.125	1.12 (0.45,2.8)	0.802
< 10	3.0 (0.84,6.56)	0.006	1.43 (0.6,3.4)	0.417
Gleason score				
8–10	Ref		Ref	
7	1.25 (0.86,1.82)	0.238	1.01 (0.66,1.53)	0.966
6	2.91 (1.81,4.69)	< 0.001	1.86 (1.08,3.22)	0.026
Clinical T stage				
3–4	Ref		Ref	
2c	3.02 (1.15,7.93)	0.025	2.51 (0.84,7.5)	0.099
1-2b	4.43 (1.79,10.99)	0.001	1.73 (0.6,4.99)	0.307
D’Amico classification				
High	Ref		Ref	
Intermediate	1.4 (1.03,1.91)	0.03	0.69 (0.46,1.03)	0.071
Low	2.36 (1.6,3.48)	< 0.001	0.73 (0.42,1.24)	0.245
Neoadjuvant treatment				
None	Ref		Ref	
ADT	0.78 (0.49,1.24)	0.296	1.67 (0.94,2.98)	0.082
Anti-A	0.55 (0.33,0.92)	0.022	0.64 (0.36,1.15)	0.139
NACHT	0.48 (0.3,0.76)	0.002	1.16 (0.64,2.13)	0.623
Prostate volume (cm^3^)				
< 35	Ref	0.569		
> = 35	0.89 (0.6,1.33)			

### Clinical characteristics (adjusted cohort)

To properly compare the oncological and functional outcomes between NS and non-NS RARP, the demographics and pathologic characteristics of the cohort matched using propensity and stratified by the nerve approach, are shown in Table 3. After propensity matching, 476 (50%) patients were treated with NS RARP and 476 (50%) with non-NS RARP; no significant differences were recorded between these two groups in terms of age, PSA, clinical T stage, Gleason score, D’Amico risk classification, BMI, prostate volume, neoadjuvant hormonal treatment, and preoperative potency (all *p* > 0.05).

**Table 3:** Clinicopathologic characteristics of 972 patients with prostate cancer treated with RARP, comparing NS RARP and non-NS RARP cohorts after propensity matching.

Variable	Overall after propensity score-matched patients (n = 952, 100%)	Nerve-sparing (n = 476, 50%)	Non nerve-sparing (n = 476, 50%)	*p*-value
Age, median, (IQR)	69 (64,73)	69 (64,73)	69 (65,73)	0.997
Age, years				1
< 65	225 (23.6)	113 (23.7)	112 (23.5)	
> = 65	727 (76.4)	363 (76.2)	364 (6.5)	
PSA (ng/mL), median, (IQR)	8.2 (5.7,12.2)	7.9 (5.5,12.2)	8.4 (5.9,12.4)	0.098
PSA (ng/mL)				0.055
< 10	608 (63.9)	319 (67)	303 (63.7)	
10–19.99	234 (24.6)	115 (24.2)	115 (24.2)	
> = 20	110 (11.5)	42 (8.8)	58 (12.1)	
Clinical T stage				0.063
1-2b	103 (10.8)	52 (10.9)	51 (10.7)	
2c	488 (51.3)	226 (47.5)	262 (55)	
3–4	361 (37.9)	198 (41.6)	163 (34.3)	
Gleason score				0.619
6	97 (10.2)	46 (9.7)	51 (10.7)	
7	513 (53.9)	251 (52.7)	262 (55)	
8–10	342 (35.9)	179 (37.6)	163 (34.3)	
D’Amico risk classification				0.223
High	592 (62.2)	283 (59.5)	309 (64.9)	
Intermediate	325 (34.1)	174 (36.6)	151 (31.7)	
Low	35 (3.7)	19 (39.9)	16 (3.4)	
BMI (kg/m^2^), median, (IQR)	23.6 (21.8,25.6)	23.6 (21.8,25.5)	23.7 (21.8,25.7)	0.731
BMI group (kg/m^2^)				0.872
< 18.5	13 (1.4)	6 (1.3)	7 (1.5)	
18.5–22.9	352 (37)	182 (38.2)	170 (35.7)	
23–24.9	258 (27.1)	129 (27.1)	129 (27.1)	
25–29.9	301 (31.6)	147 (30.9)	154 (32.3)	
> = 30	28 (2.9)	12 (2.5)	16 (3.4)	
Prostate volume preoperative group (cm^3^)				0.436
< 35	527 (55.4)	270 (56.7)	237 (49.8)	
> = 35	425 (44.6)	206 (43.3)	239 (50.2)	
Neoadjuvant treatment				0.056
None	476 (50)	250 (52.5)	226 (44.5)	
ADT	140 (14.7)	69 (14.5)	71 (14.9)	
Anti-A	106 (11.1)	59 (12.4)	47 (9.9)	
NACHT	230 (24.2)	98 (20.6)	132 (27.7)	
Preoperative Potency				0.477
No erection	454 (47.7)	227 (47.7)	227 (47.7)	
Erection	323 (33.9)	155 (32.6)	168 (35.3)	
Penetration	175 (18.4)	94 (19.7)	81 (17)	

### Pentafecta achievement and clinical outcome of RARP comparison between NS and non-NS after propensity matching

**Figure 2:**
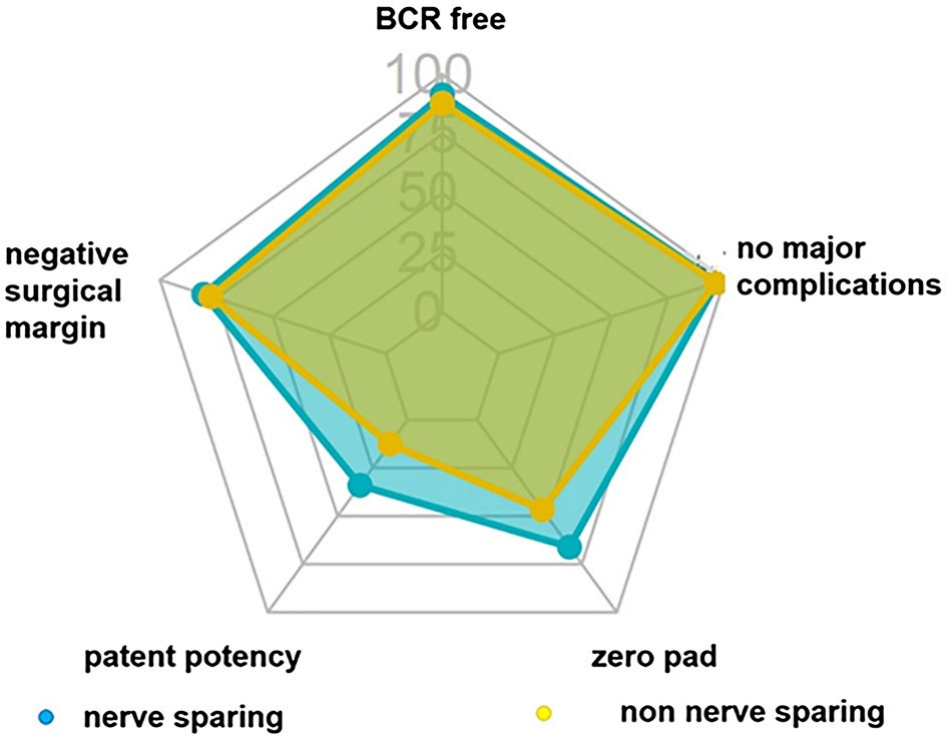
Achievement of pentafecta outcome after RARP between NS and non-NS approach.

Multivariate analysis demonstrated that the NS approach in RARP was associated with pentafecta achievement, as shown in Table 2. After a decrease in the risk of bias, propensity matching was performed, and important clinical outcomes were compared between these two groups (Table 4). We found that the NS group had better outcomes in terms of operation and console time, postoperative potency, pad-free status, early continence pad-free status in the first 3 months, trifecta achievement (no BCR, continence, and potency), quadrifecta achievement (no complications, continence, negative SM, and no BCR), and pentafecta achievement, as described. However, the rates of BCR-free status, negative SM, and absence of major postoperative complications were not significantly different. Moreover, we observed the results for each important factor in the pentafecta in both groups (Fig. 2).

**Table 4:** RARP comparison of NS and non-NS after propensity matching.

Outcomes	Overall (n = 952, 100%)	Nerve-sparing (n = 476, 50%)	Non nerve-sparing (n = 476, 50%)	*p*-value
Operative time, min, (IQR)	174 (148,209)	166 (144,196)	180 (158,219)	< 0.001
Console time, min, (IQR)	126 (104,156)	120 (100,146)	127 (108,156)	< 0.001
Blood loss, mL, (IQR)	220 (123,352)	203.5 (114,348)	222 (134,346)	0.412
Biochemical recurrence-free	844 (88.7)	426 (89.5)	418 (87.9)	0.472
Negative surgical margin	756 (78.6)	380 (79.8)	368 (77.4)	0.392
Postoperative potency				< 0.001
No erection	768 (79.8)	343 (72.1)	415 (87.2)	
Erection	166 (17.3)	111 (23.3)	55 (11.5)	
Penetrate	28 (2.9)	22 (4.6)	6 (1.3)	
Pad free (0 pad per day)	526 (55.3)	304 (63.8)	222 (46.7)	< 0.001
Absence of major perioperative complications	920 (96.7)	462 (97.1)	458 (96.3)	0.58
Early pad free at 3 month	143 (15)	90 (18.9)	53 (11.1)	0.001
Trifecta achievement	384 (40.3)	223 (46.8)	159 (33.4)	< 0.001
Qaudrifecta achievement	93 (9.8)	73 (15.3)	20 (4.2)	< 0.001
Pentafecta achievement	91 (9.6)	72 (15.1)	19 (4)	< 0.001

## Discussion

To date, no published randomized trial has conducted a comprehensive comparison between NS and non-NS RARP for localized or locally advanced PCa. However, our previous study focused on selected high-risk PCa cases and revealed that the NS technique yielded equivalent oncological outcomes while improving urinary continence compared with the non-NS RARP group^15^. Furthermore, our retrospective study was the first to compare the achievement of pentafecta outcomes under these conditions. It is important to note that retrospective studies inherently possess limitations such as selection bias, variations in treatment protocols, and unclear outcome definitions. To address these concerns, our retrospective study implemented a PS-matched method, strictly controlled patient enrollment, and employed clearly defined outcome measures, all aimed at providing valuable guidance for decision-making. Our research findings demonstrate that NS RARP yields superior pentafecta outcomes compared with non-NS RARP, particularly in terms of postoperative urinary continence and potency. Furthermore, multivariate analysis established that the NS approach significantly increased the likelihood of achieving pentafecta outcomes in patients diagnosed with localized prostate cancer. These results offer invaluable clinical insights into their potential to significantly influence strategic disease management.

The attainment of pentafecta outcomes in NS RARP represents the ultimate objective of the surgical management of localized PCa. By conducting a comprehensive evaluation of the surgical, functional, and oncologic outcomes, complications, and patient satisfaction, we gained a holistic understanding of the success of the procedure. In this discussion, we examine each domain of the pentafecta outcome and explore the implications of NS RARP. Specifically, we focus on urinary continence, potency recovery, freedom from BCR, absence of major perioperative complications within 90 days, and negative SM, as these components constitute the pentafecta and represent the most desired outcomes following RP. These components are of paramount importance when assessing intermediate- and long-term outcomes in patients who have undergone RP for PCa management. Traditionally, RP outcomes have been reported separately, with only a few studies providing information on the percentage of patients who achieved pentafecta. Among these components, potency recovery is the most challenging outcome to achieve post-surgery and is often the primary factor contributing to the failure to attain pentafecta.. Interestingly, when assessing laparoscopic radical prostatectomy (LRP) and robot-assisted laparoscopic prostatectomy (RALP) outcomes for pT3 disease cases, equivalent pentafecta rates are observed. However, it is important to note that 'tetrafecta' outcomes, excluding potency recovery from postoperative expectations, exhibit similarity between these two techniques. These findings underscore the complex interplay of surgical approach and patient expectations in achieving optimal outcomes in prostate cancer management^16^.

Previous studies have employed various endpoints to assess postoperative pentafecta attainment. Multiple factors are considered when evaluating erectile function, such as partial recovery, satisfactory rigidity, ability to engage in sexual intercourse, and overall sexual satisfaction^17^. In numerous studies, potency has been defined as achieving an erection sufficient for intercourse (ESI), with or without the use of a PDE5 inhibitor. However, in the present study, we expanded the definition of potency beyond sexual intercourse to encompass sexual activity, including masturbation.

A previous study revealed that Japanese patients with　PCa　exhibit a low occurrence of sexual intercourse following RARP. However, they engage in sexual activities, particularly masturbation, at a relatively high frequency. It has been previously reported that the Japanese population has a low rate of sexual intercourse^18^, making it challenging to accurately assess sexual function using the IIEF (International Index of Erectile Function), which relies on sexual intercourse as a criterion. Additionally, in our study, we considered potency to be present if the patient experienced erections or penetration during sexual intercourse. Notably, 40% of the participants in our study lacked preoperative potency, which influenced the achievement of the pentafecta outcomes. Numerous studies have defined continence as achieving a pad-free status^19,20^. In addition, the use of zero-pads has been acknowledged as a satisfactory measure for assessing continence in other studies. BCR was evaluated using sequential serum PSA measurements, with the commonly adopted definition of BCR as a PSA level exceeding 0.2 ng/mL^2,21^.

A previous longitudinal study conducted among Japanese patients with PCa revealed a significant decline in sexual function after RP^22^. However, in our study, we expanded the definition of potency beyond sexual intercourse to include sexual activity such as masturbation. Notably, this definition may be specific to our study. Furthermore, a previous investigation revealed that Japanese men were less likely to be concerned about their sexual function despite experiencing a lower frequency of erections than their American counterparts. These reports emphasize the importance of considering fundamental and cultural differences when evaluating erectile dysfunction (ED)^23^. In our study, longitudinal analysis revealed that pentafecta rates remained consistently lower than trifecta rates. To provide a more comprehensive evaluation of RP outcomes, we proposed adding postoperative complications and histological margin status to existing trifecta outcomes^24^. The inclusion of these two additional components in the pentafecta resulted in a relatively low overall rate of 9.6%. However, the NS group exhibited a significantly higher rate than the non-NS group (15.1% vs. 4%, *p* < 0.001). It is important to highlight that these rates are lower than those reported in a previous study by Karagiotis et al.^10^, in which the stable quality of life (QoL) rates for cancer of the prostate risk assessment LR vs. HR and pentafecta were 30%, 26%, and 30%, respectively.

Through a more detailed examination of 603 patients who underwent NS RARP and were assessed for pentafecta outcomes based on the risk groups using the D'Amico classification, Affreri et al. revealed notable shifts in the attainment of pentafecta outcomes. Specifically, patients classified as low-risk experienced a decrease from 33 to 20%, whereas those in the intermediate-risk group experienced an increase from 52 to 62%. Patients categorized as high-risk demonstrated a modest increase from 10 to 13%. Overall, the proportion of patients who achieved Pentafecta increased from 38 to 44%. Notably, the primary reason for not achieving pentafecta was the absence of postoperative potency, which accounted for 71% of the cases. BCR strongly influenced the achievement of pentafecta in the high-risk group (61%) but had a lesser impact in the intermediate- (24%) and low-risk (30%) groups^25^.

In another study involving 566 patients who underwent RARP, the reported rates of trifecta and pentafecta were 73.9% and 64.1%, respectively^26^. Additionally, a previous study that specifically focused on bilateral nerve bundle sparing in 230 patients who underwent RARP demonstrated a pentafecta rate of 60.4% (139/230)^27^. It is widely recognized that pentafecta outcomes align more accurately with patient expectations after surgery for PCa^2^. Based on our Pentafecta results, further refinement of the surgical technique in RARP is necessary to enhance Pentafecta outcomes.

Despite the strengths of this study, it is important to acknowledge its limitations as well. The retrospective design and inherent risk of bias associated with retrospective studies are notable limitations. Furthermore, the study's short follow-up duration in some cases and its single-center nature, involving a limited number of surgeons, introduce the possibility of overlooking unknown confounding factors influenced by surgeon experience, judgment of indications for NS, population differences, and unmeasured variables.

This study indicated that NS, especially BNS and UNSs, were associated with increased pentafecta achievement. Furthermore, this series reflects the optimal outcomes for high-volume expert surgeons. However, to the best of our knowledge, this is one of the largest cohorts in the literature comparing outcomes in patients with similar perioperative characteristics (balanced with PS) and full access to the gold standard treatments for PCa.

In conclusion, the pentafecta outcome of NS RARP represents an amalgamation of surgical, functional, oncologic, and patient-centered achievements. Through ongoing research, technological advancements, and multidisciplinary collaborations, NS RARP continues to evolve, offering improved outcomes for patients with localized PCa. Addressing the challenges in each domain of the pentafecta and striving for individualized treatment approaches will pave the way for further enhancements in NS RARP and ultimately optimize patient outcomes and satisfaction.

## Conclusion

NS RARP showed superior pentafecta outcomes compared to non-NS RARP, with especially significant difference in postoperative urinary continence or erectile function. Multivariate analysis revealed that the NS approach increased the pentafecta achievement rate in patients with localized PCa who were selected using propensity score matching. Therefore, we propose a more precise approach for determining NS outcomes. We believe that the pentafecta outcomes reflect patient expectations after RP more accurately. This approach may be beneficial and should be used when counseling patients with clinically localized PCa.

## Data Availability

The datasets used and/or analysed during the current study are available from the corresponding author on reasonable request.

## References

[1] Sung H, Ferlay J, Siegel RL, Laversanne M, Soerjomataram I, Jemal A, Bray F (2021). Global cancer statistics 2020: GLOBOCAN estimates of incidence and mortality worldwide for 36 cancers in 185 countries. CA Cancer J Clin..

[2] Patel VR, Sivaraman A, Coelho RF, Chauhan S, Palmer KJ, Orvieto MA, Camacho I, Coughlin G, Rocco B (2011). Pentafecta: A new concept for reporting outcomes of robot-assisted laparoscopic radical prostatectomy. Eur. Urol..

[3] Reeves F, Preece P, Kapoor J, Everaerts W, Murphy DG, Corcoran NM, Costello AJ (2015). Preservation of the neurovascular bundles is associated with improved time to continence after radical prostatectomy but not long-term continence rates: Results of a systematic review and meta-analysis. Eur. Urol..

[4] Suardi N, Moschini M, Gallina A, Gandaglia G, Abdollah F, Capitanio U, Bianchi M, Tutolo M, Passoni N, Salonia A, Hedlund P, Rigatti P, Montorsi F, Briganti A (2013). Nerve-sparing approach during radical prostatectomy is strongly associated with the rate of postoperative urinary continence recovery. BJU Int..

[5] Haga N, Miyazaki T, Tsubouchi K, Okabe Y, Shibayama K, Emoto D, Matsuoka W, Maruta H, Aoyagi C, Matsuzaki H, Irie S, Nakamura N, Matsuoka H (2021). Comprehensive approach for preserving cavernous nerves and erectile function after radical prostatectomy in the era of robotic surgery. Int. J. Urol..

[6] Druskin SC, Liu JJ, Young A, Feng Z, Dianat SS, Ludwig WW, Trock BJ, Macura KJ, Pavlovich CP (2017). Prostate MRI prior to radical prostatectomy: effects on nerve sparing and pathological margin status. Res. Rep. Urol..

[7] Zhang L, Wu B, Zha Z, Zhao H, Yuan J, Jiang Y, Yang W (2018). Surgical margin status and its impact on prostate cancer prognosis after radical prostatectomy: a meta-analysis. World J. Urol..

[8] Yossepowitch O, Briganti A, Eastham JA, Epstein J, Graefen M, Montironi R, Touijer K (2014). Positive surgical margins after radical prostatectomy: A systematic review and contemporary update. Eur. Urol..

[9] Wojtarowicz M, Przepiera A, Lemiński A, Gołąb A, Słojewski M (2023). Assessment of the impact of pentafecta parameters affecting the quality of life of patients undergoing laparoscopic radical prostatectomy. Int. J. Environ. Res. Public Health.

[10] Karagiotis T, Witt JH, Jankowski T, Mendrek M, Wagner C, Schuette A, Liakos N, Rachubinski P, Urbanova K, Oelke M, Kachanov M, Leyh-Bannurah SR (2022). Two-year quality of life after robot-assisted radical prostatectomy according to pentafecta criteria and cancer of the prostate risk assessment (CAPRA-S). Sci. Rep..

[11] D'Amico AV, Whittington R, Malkowicz SB, Schultz D, Blank K, Broderick GA, Tomaszewski JE, Renshaw AA, Kaplan I, Beard CJ, Wein A (1998). Biochemical outcome after radical prostatectomy, external beam radiation therapy, or interstitial radiation therapy for clinically localized prostate cancer. JAMA.

[12] Sobin, L. H. & Fleming, I. D. TNM Classification of malignant tumors, fifth edition (1997). Union Internationale Contre le Cancer and the American Joint Committee on Cancer. *Cancer ***80**(9),1803–4 (1997). 10.1002/(sici)1097-0142(19971101)80:9<1803::aid-cncr16>3.0.co;2-9. PMID: 9351551. 10.1002/(sici)1097-0142(19971101)80:9<1803::aid-cncr16>3.0.co;2-9 9351551

[13] Dindo D, Demartines N, Clavien PA (2004). Classification of surgical complications: a new proposal with evaluation in a cohort of 6336 patients and results of a survey. Ann. Surg..

[14] Lu YC, Huang CY, Cheng CH, Huang KH, Lu YC, Chow PM, Chang YK, Pu YS, Chen CH, Lu SL, Lan KH, Jaw FS, Chen PL, Hong JH (2022). Propensity score matching analysis comparing radical prostatectomy and radiotherapy with androgen deprivation therapy in locally advanced prostate cancer. Sci. Rep..

[15] Takahara K, Sumitomo M, Fukaya K, Jyoudai T, Nishino M, Hikichi M, Zennami K, Nukaya T, Ichino M, Fukami N, Sasaki H, Kusaka M, Shiroki R (2019). Clinical and oncological outcomes of robot-assisted radical prostatectomy with nerve sparing vs. non-nerve sparing for high-risk prostate cancer cases. Oncol. Lett..

[16] Asimakopoulos, A.D., Miano, R., Di Lorenzo, N., Spera, E., Vespasiani, G. & Mugnier, C. Laparoscopic versus robot-assisted bilateral nerve-sparing radical prostatectomy: comparison of pentafecta rates for a single surgeon. *Surg. Endosc.* **27**(11), 4297–304 (2013). 10.1007/s00464-013-3046-9. Epub 2013 Jun 27. Erratum in: Surg. Endosc. 2013 Oct;27(10):3955. PMID: 23807752. 10.1007/s00464-013-3046-9 23807752

[17] Antebi E, Eldefrawy A, Katkoori D, Soloway CT, Manoharan M, Soloway MS (2011). Oncological and functional outcomes following open radical prostatectomy: How patients may achieve the "Trifecta"?. Int. Braz. J. Urol..

[18] Namiki S, Kwan L, Kagawa-Singer M, Saito S, Terai A, Satoh T, Baba S, Arai Y, Litwin MS (2008). Sexual function reported by Japanese and American men. J. Urol..

[19] Xylinas E, Durand X, Ploussard G, Campeggi A, Allory Y, Vordos D, Hoznek A, Abbou CC, de la Taille A, Salomon L (2013). Evaluation of combined oncologic and functional outcomes after robotic-assisted laparoscopic extraperitoneal radical prostatectomy: trifecta rate of achieving continence, potency and cancer control. Urol. Oncol..

[20] Novara G, Ficarra V, D'Elia C, Secco S, Cavalleri S, Artibani W (2011). Trifecta outcomes after robot-assisted laparoscopic radical prostatectomy. BJU Int..

[21] Ficarra V, Sooriakumaran P, Novara G, Schatloff O, Briganti A, Van der Poel H, Montorsi F, Patel V, Tewari A, Mottrie A (2012). Systematic review of methods for reporting combined outcomes after radical prostatectomy and proposal of a novel system: The survival, continence, and potency (SCP) classification. Eur. Urol..

[22] Inoue S, Shiina H, Hiraoka T, Wake K, Sumura M, Honda S, Urakami S, Igawa M, Usui T (2009). Five-year longitudinal effect of radical perineal prostatectomy on health-related quality of life in Japanese men, using general and disease-specific measures. BJU Int..

[23] Namiki S, Arai Y (2010). Health-related quality of life in men with localized prostate cancer. Int. J. Urol..

[24] Patel VR, Abdul-Muhsin HM, Schatloff O, Coelho RF, Valero R, Ko YH, Sivaraman A, Palmer KJ, Chauhan S (2011). Critical review of 'pentafecta' outcomes after robot-assisted laparoscopic prostatectomy in high-volume centres. BJU Int..

[25] Afferi L, Moschini M, Baumeister P, Zamboni S, Cornelius J, Ineichen G, Mattei A, Mordasini L (2021). Trends in risk-group distribution and Pentafecta outcomes in patients treated with nerve-sparing, robot-assisted radical prostatectomy: A 10-year low-intermediate volume single-center experience. World J. Urol..

[26] Jazayeri SB, Weissman B, Samadi DB (2018). Outcomes following robotic-assisted laparoscopic prostatectomy: Pentafecta and Trifecta achievements. Minerva Urol. Nefrol..

[27] Ou YC, Yang CK, Kang HM, Chang KS, Wang J, Hung SW, Tung MC, Tewari AK, Patel VR (2015). Pentafecta outcomes of 230 cases of robotic-assisted radical prostatectomy with bilateral neurovascular bundle preservation. Anticancer. Res..

